# Deconvolving bulk proteomics data via deep adaptation network

**DOI:** 10.1093/bib/bbaf631.032

**Published:** 2025-12-12

**Authors:** Zequan Xie, Jihong Tang, Jiguang Wang

**Affiliations:** Division of Life Science, Department of Computer Science and Engineering, The Hong Kong University of Science and Technology, Hong Kong SAR, China; Division of Life Science, Department of Chemical and Biological Engineering, State Key Laboratory of Nervous System Disorders, The Hong Kong University of Science and Technology, Hong Kong SAR, China; Division of Life Science, Department of Chemical and Biological Engineering, State Key Laboratory of Nervous System Disorders, The Hong Kong University of Science and Technology, Hong Kong SAR, China

## Abstract

Bulk proteomic assays measure average protein abundances across heterogeneous tissues, while deconvolution algorithms attempt to estimate the proportions of distinct cell types from such data. However, existing proteomics deconvolution methods often underperform when the same cell type has different proportion distributions in training and target data. To bridge this gap, we propose Deconv-DAN, a novel machine learning framework for bulk proteomic deconvolution. Deconv-DAN begins by generating a diverse set of pseudo-bulk training samples where it draws cell type proportions from a heavy-tailed distribution, guided by a k-nearest neighbors classifier which estimates the most abundant cell type in each target sample. Next, a Deep Adaptation Network [1] combining prediction loss and domain adaptation loss is trained. We evaluate Deconv-DAN on both simulated data (specifically designed to mimic mismatched proportion distributions) and real bulk proteomics datasets. Across both settings, Deconv-DAN yields superior deconvolution accuracy compared to existing methods. We further demonstrate its adaptability by applying it to DNA methylation deconvolution, where it attains competitive performance. Deconv-DAN therefore offers a solution for bulk deconvolution in proteomics and beyond, particularly in scenarios with mismatched training and target cell composition distributions. We expect Deconv-DAN to be useful in revealing heterogeneity of different tissues and tumor microenvironment.

**References:**

[1] Long, M, et al. ‘Learning transferable features with deep adaptation networks.’ In: Proceedings of the 32nd International Conference on Machine Learning. 2015, 97–105.

